# Assessment of Knowledge and Barriers to Colon Cancer Screening Among the General Public in the Qassim Region

**DOI:** 10.7759/cureus.82047

**Published:** 2025-04-10

**Authors:** Abdullah Mohammed, Mayadah A Alawaji, Amal Bayen Alharbi, Raghad Abdullah Alkhuwaiter, Raghad Mansour Alwehaibi, Asma Abdullah Alsohaibani, Reema Ali Almuzaini, Norah Hamad Alabdullatif, Nawaf Almutairi, Edward Mugambi Ireri

**Affiliations:** 1 Gastroenterology, King Fahad Specialist Hospital, Buraydah, SAU; 2 Faculty of Medicine, Qassim University, Qassim, SAU; 3 Faculty of Medicine, King Fahad Specialist Hospital, Buraydah, SAU; 4 Data Science and Analytics, Smart Health EQUAS Consultants Limited Company, Nairobi, KEN

**Keywords:** colonoscopy, colorectal cancer screening, fecal occult blood test, saudi arabia, symptom-based screening

## Abstract

Background: Colorectal cancer (CRC) prevalence in Saudi Arabia has been increasing in recent years. To enhance the uptake of preventive CRC screening services, it is important to understand the individual enablers and barriers associated with screening.

Materials and methods: A descriptive cross-sectional study employing convenience sampling was conducted using a self-administered online survey among 500 adult participants residing in the Qassim Region. The data collection took place between July 30, 2024, and September 1, 2024. Logistic regression was performed using R programming (R Foundation for Statistical Computing, Vienna, Austria), while Python (Python Software Foundation, Wilmington, DE) was utilized for graph generation.

Results: Adults' knowledge of CRC screening was significantly influenced by family history (AdjOR = 0.152; 99% CI: 0.056-0.393), symptom-based screening (AdjOR = 1.963; 95% CI: 1.045-3.730), and discussions with health promoters (AdjOR = 35.25; 99% CI: 15.36-90.84). Barriers to colorectal screening were significantly influenced by the perception that CRC is not a serious health threat (AdjOR = 2.059; 99% CI: 1.252-3.389) and a lack of transportation (AdjOR = 1.589; 95% CI: 1.017-2.477). Past negative screening experiences were significant barriers to colonoscopy (AdjOR = 2.818; 99% CI: 1.751-4.640), while the belief that the fecal occult blood test was not important (AdjOR = 2.147; 99% CI: 1.374-3.423) increased the likelihood of CRC screening.

Conclusion: Notable information gaps and low awareness of CRC screening persist. Transportation challenges and past negative experiences with colonoscopy services discourage individuals from seeking preventive care. The Ministry of Health must address perceptions of screening to promote behavioral change and dispel misconceptions by providing psychological support, public education, and financial assistance to reduce barriers.

## Introduction

Colorectal cancer (CRC) is the second most frequent cancer in Saudi Arabia, being the most prevalent in men (10.6%) and the third most common in women (8.9%). In 2004, the World Health Organization reported that 8.3% of Saudi Arabian deaths were related to CRC. In 2022, the overall rate of CRC in Saudi Arabia was 13.3%, with men recording 17.2% and women 9.1% [1].

Regular screening can contribute to early discovery and substantially improve patient prognosis, with five-year survival rates for early-stage CRC being over 90% [2]. CRC screening involves detecting early signs of cancer in individuals without symptoms. The goal is to identify precancerous polyps or early-stage cancer when treatment is more effective and survival rates are higher.

Raising CRC screening rates can lower morbidity and mortality from this highly avoidable illness, making it a key healthcare priority. Routine screening procedures can identify precancerous polyps or early-stage malignancies, enabling prompt intervention and treatment. Examples of these procedures are colonoscopy, sigmoidoscopy, and fecal occult blood tests [3]. The fecal immunochemical test (FIT) is a noninvasive stool test that detects hidden blood, which may indicate polyps or cancer. It is commonly used as a first-line screening method in Saudi Arabia and is performed annually. Colonoscopy involves inserting a flexible camera into the rectum to examine the entire colon. If the results are normal, it is done every 10 years. Flexible sigmoidoscopy examines only the lower part of the colon and is performed every 5-10 years. CT colonography, or virtual colonoscopy, uses CT scans to visualize the colon and is recommended every five years.

Screening for CRC is recommended to begin at age 45 for average-risk individuals, in accordance with Saudi National Guidelines. However, individuals at high risk may require earlier screening. This includes those with a family history of CRC or adenomatous polyps, genetic syndromes like familial adenomatous polyposis or Lynch syndrome, and a personal history of inflammatory bowel disease. For these individuals, screening may start at age 40 or 10 years earlier than the age at which the earliest diagnosed relative was affected. The average-risk population includes men and women aged 45-75 with no symptoms or family history of CRC.

To develop effective measures that increase screening uptake, it is essential first to understand the elements that influence an individual's knowledge, awareness, and opinions regarding CRC and its screening. Many obstacles to CRC screening have been observed in previous studies, such as fear, inadequate awareness [4], cultural preconceptions [5], embarrassment associated with screening procedures [6], low socioeconomic status [7], insufficient routine health check-up [8], and lack of physician recommendation [9].

Reduced capacity to acquire and interpret health information and a lower propensity to engage in preventative medical practices, such as CRC screening, are linked to low health literacy [10]. Since the disease is more widespread in Saudi Arabia than other malignancies, screening strategies specific to the setting need to be implemented [11]. The Saudi guidelines for CRC screening have been utilized opportunistically since they were released [11]. Significant organizational and infrastructure resources need to be addressed before a nationwide program can be implemented [12]. Furthermore, there are no official cost-effectiveness studies on nationwide screening, as evidenced by the age-adjusted rate for neoplasia in general [13].

Different demographic areas, as well as various healthcare facilities and communities, may have distinct, unique barriers and facilitators. The purpose of this proposed study is to thoroughly evaluate the population in the Qassim region's present state of knowledge and awareness regarding the importance of CRC screening and the method. It will also pinpoint the main obstacles and enablers of CRC screening in this community. The results of this study will have a direct impact on developing focused educational initiatives and other interventions aimed at raising CRC screening rates and, ultimately, lowering the incidence of this avoidable illness.

Therefore, this paper assesses the public understanding of CRC screening in the Qassim region and points out the possible obstacles to involvement in screening campaigns.

## Materials and methods

Study design

A descriptive cross-sectional study was conducted using a self-administered online survey via WhatsApp and Twitter (the X-Platform) among 500 adults (over 18 years old) who are residents of Qassim. The study took place from July 30, 2024, to September 1, 2024.

Study population

The study used convenience sampling, and the patients were recruited based on their availability and willingness to participate in the research. This is why, despite the minimum sample size for the study having been computed as 384, a total of 500 patients gave consent to participate in the study. The Qassim region is not densely populated, making a sample of 500 individuals relatively large and diverse within this context.

The study's target population included adults, a typically employed and busy demographic more likely to respond to online questionnaires at their convenience rather than commit to scheduled interviews. Reaching and engaging this population through direct interviews would have been highly challenging, especially without a centralized location or institutional access. Therefore, an online survey enabled us to efficiently and effectively reach our target population while meeting our study goals within the available resources and timeframe.

Ethical statement

Ethical approval was sought from the Qassim University Institutional Review Board (60746926). Informed consent was sought from the study participants: written signatures for those who could read and write, and thumbprints for those who could not read and write.

The study tool

The questionnaire comprised four sections: demographic information; an assessment of patient knowledge about CRC screening; an assessment of previous experience with screening; and an assessment of the barriers to CRC screening.

The demographic section collected data on patients' age, gender, and level of education, all of which were categorical in nature and treated as confounders in the study (see the questionnaire in the Appendix). Questions 1-10 assessed patients' knowledge of CRC screening and were treated as predictors, with categorical responses. Question 11 was categorical and was treated as the outcome variable, "Colorectal (CR) Screening." Questions 12-14 assessed previous screening experiences and were also categorical. Questions 15-28 assessed barriers to CRC screening, and their responses were categorical. Questions 29-34 examined barriers to fecal occult blood testing, with responses also measured categorically.

The questionnaire was in the Arabic language, which was translated to English to allow the statistician to analyze the data with ease, thus maintaining data integrity.

Data analysis and graphic presentation

Data analysis and graphical presentation were performed using R (R Foundation for Statistical Computing, Vienna, Austria) and Python (Python Software Foundation, Wilmington, DE). The odds ratios for the risk factors were estimated using logistic regression, and p values less than 0.05 were considered significant.

**Figure 1:**
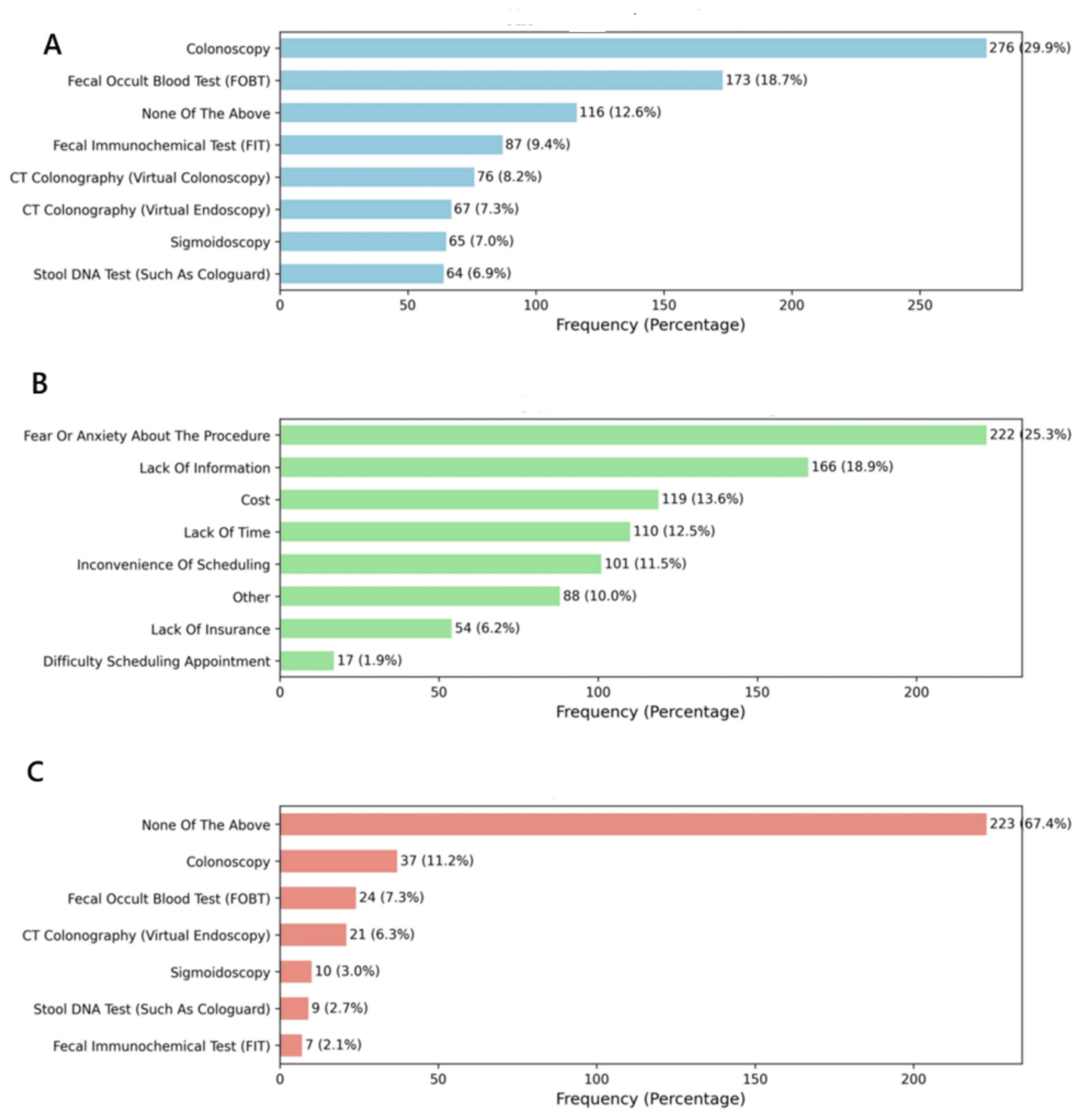
A descriptive analysis of the (A) knowledge, (B) barriers, and (C) methods of colorectal cancer screening FOBT: fecal occult blood test; FIT: fecal immunochemical test

Three questions, knowledge, barriers, and screening methods, were assessed using multiple responses. The graphical presentation was conducted using Python packages, specifically Pandas and Counter from the collections module, which was used to summarize the multiple responses in each column. The responses were separated using semicolons and split accordingly. The summary was then converted into a DataFrame. Extra spaces and hidden special characters were removed from the responses. Each response was formatted to start with a capital letter, spelling errors were corrected, and duplicate entries were deleted. Standardization of synonymous responses was performed, followed by ensuring that proper medical terminologies were capitalized. Variations in some phrases were also handled through advanced cleaning and standardization. The code generated a clean DataFrame with three columns, Knowledge, Barriers, and Methods, which enabled the plotting of bar graphs using the Seaborn and Matplotlib packages (Figure 1).

## Results

The descriptive statistics provided in Table 1 present only significant measures of associations obtained after running chi-square tests with CR Screening as the dependent variable. The results are presented in the form of frequencies and percentages. The percentages presented are column percentages (between percentages), which represent the proportion of individuals within the screening or no screening group.

**Table 1:** Descriptives and measures of associations The p value for education level should be interpreted with caution, as 41.7% of the five cells have an expected count below five FOBT: fecal occult blood test

Variable	Characteristic	Screening	No screening	p value
Age	<20	21 (39.6%)	197 (44.1%)	0.006
	21-30	0 (0%)	72 (16.1%)	
	31-40	9 (17.0%)	60 (13.4%)	
	41-50	12 (22.6%)	77 (17.2%)	
	51-60	8 (15.1%)	32 (7.2%)	
	>61	3 (5.7%)	9 (2%)	
Gender	Female	40 (8%)	373 (74.6%)	0.148
	Male	13 (2.6%)	74 (14.8%)	
Education	Not educated	3 (0.6%)	1 (0.2%)	<0.001
	Primary education	3 (0.6%)	3 (0.6%)	
	Intermediate education	10 (2%)	4 (0.8%)	
	Secondary education	63 (12.9%)	9 (1.8%)	
	Bachelor	353 (70.6%)	31 (6.2%)	
	Masters	15 (3%)	5 (1%)	
Knowledge of colon rectal screening				
About early screening	Yes	42 (79.2%)	239 (53.5%)	<0.001
	No	11 (20.8%)	208 (46.5%)	
Recommended screening age	40 years	26 (49.1%)	152 (34%)	<0.001
	45 years	11 (20.8%)	77 (17.2%)	
	50 years	12 (22.6%)	79 (17.7%)	
	55 years	0 (0.0%)	16 (3.6%)	
	60 years	2 (3.8%)	24 (5.4%)	
	Not sure	2 (3.8%)	99 (22.1%)	
Screening frequency	Every year	18 (34.0%)	85 (19%)	0.007
	Every 3 years	16 (30.2%)	90 (20.1%)	
	Every 5 years	11 (20.8%)	93 (20.8%)	
	Every 10 years	2 (3.8%)	47 (10.5%)	
	Only if a symptom appears	5 (9.4%)	73 (16.3%)	
	Not sure	1 (1.9%)	59 (13.2%)	
Family history	Yes	42 (79.2%)	358 (80.1%)	<0.001
	No	10 (18.9%)	24 (5.4%)	
	I am not sure	1 (1.9%)	65 (14.5%)	
Symptom-based screening	Yes	34 (64.2%)	199 (44.5%)	0.025
	No	15 (28.3%)	191 (42.7%)	
	I am not sure	4 (7.5%)	57 (12.8%)	
Health promoter discussion	Yes	40 (75.5%)	37 (8.3%)	<0.001
	No	13 (24.5%)	410 (91.7%)	
Where to access screening	Yes	41 (77.4%)	141 (31.5%)	<0.001
	No	7 (13.2%)	222 (49.7%)	
	I am not sure	5 (9.4%)	84 (18.8%)	
Barriers to colon rectal screening				
Is not mandatory	Disagree	12 (22.6%)	106 (23.7%)	0.031
	Neutral	11 (20.8%)	165 (36.9%)	
	Agree	30 (56.6%)	176 (39.4%)	
Not effective	Disagree	19 (35.8%)	292 (65.3%)	<0.001
	Neutral	16 (30.2%)	120 (26.8%)	
	Agree	18 (34%)	35 (7.8%)	
Not a serious health threat	Disagree	19 (35.8%)	297 (66.4%)	<0.001
	Neutral	12 (22.6%)	117 (26.2%)	
	Agree	22 (41.5%)	33 (7.4%)	
Lack transportation	Disagree	18 (34%)	267 (59.7%)	<0.001
	Neutral	13 (24.5%)	130 (29.1%)	
	Agree	22 (41.5%)	50 (11.2%)	
Colonoscopy				
Takes a lot of time	Disagree	10 (18.9%)	64 (14.3%)	<0.001
	Neutral	15 (28.3%)	257 (57.5%)	
	Agree	28 (52.8%)	126 (28.2%)	
It is not important	Disagree	16 (30.2%)	233 (52.1%)	<0.001
	Neutral	17 (32.1%)	156 (34.9%)	
	Agree	20 (37.7%)	58 (13%)	
An expensive procedure	Disagree	10 (18.9%)	106 (23.7%)	0.030
	Neutral	20 (37.7%)	224 (50.1%)	
	Agree	23 (43.4%)	117 (26.2%)	
Past bad screening experience	Disagree	12 (22.6%)	251 (56.2%)	<0.001
	Neutral	22 (41.5%)	159 (35.6%)	
	Agree	19 (35.8%)	37 (8.3%)	
FOBT				
Not important	Disagree	17 (32.1%	216 (48.3%)	<0.001
	Neutral	13 (24.5%)	182 (40.7%)	
	Agree	23 (43.4%)	49 (11%)	
An expensive procedure	Disagree	13 (24.5%)	151 (33.8%)	<0.001
	Neutral	19 (35.8%)	235 (52.6%)	
	Agree	21 (39.6%)	61 (13.6%)	
No time for FOBT	Disagree	16 (30.2%)	158 (35.3%)	0.011
	Neutral	16 (30.2%)	193 (43.2%)	
	Agree	21 (39.6%)	96 (21.5%)	
Screening discomfort	Disagree	14 (26.4%)	155 (34.7%)	0.027
	Neutral	17 (32.1%)	183 (40.9%)	
	Agree	22 (41.5%)	109 (24.4%)	

Descriptive statistics

Figure 1 presents the findings on knowledge of CR screening, barriers to CR screening, and screening methods, which are factors influencing CR screening. The figures represent questions that allowed parents to select multiple responses and display both frequencies and percentages.

Multivariate statistic

Multivariate analysis was achieved by running logistic regression on significant independent variables, which showed a significant positive association with CR screening. Overall, Table 2 showed significantly reduced odds between knowledge of CR screening and family history (AdjOR = 0.152; 99% CI: 0.056-0.393). Women showed significantly reduced odds between knowledge of CR screening and family history, the findings which were not significant among male participants (AdjOR = 0.154; 99% CI: 0.048-0.463). Symptom-based screening had an increased odds of the knowledge of CR screening in the overall model (AdjOR = 1.963; 95% CI: 1.045-3.730) and among men (AdjOR = 10.416; 95% CI: 1.663-124.95). Discussion with the health promoter had increased odds of knowledge of CR screening on the overall model (AdjOR = 35.25; 99% CI: 15.36-90.84), female participants (AdjOR = 29.837; 99% CI: 12.111-83.845), and male participants (AdjOR = 241.38; 99% CI: 15.662-21,914).

**Table 2:** Knowledge of colon rectal screening Significance levels: ^*^p < 0.1; ^**^p < 0.05; ^***^p < 0.01. The p values are enclosed in parentheses

Predictors	Overall model (1)	Females (2)	Males (3)
About early screening	-0.075 (0.881)	-0.306 (0.583)	0.644 (0.583)
Recommended screening age	-0.176 (0.217)	-0.182 (0.237)	-0.293 (0.513)
Screening frequency	-0.211 (0.170)	-0.162 (0.314)	-0.610 (0.243)
Family history	-1.881 (0.0002)^***^	-1.869 (0.002)^***^	-2.385 (0.117)
Symptom-based screening	0.674 (0.037)^**^	0.424 (0.233)	2.343 (0.028)^**^
Health promoter discussion	3.563 (<0.001)^***^	3.396 (<0.001)^***^	5.486 (0.002)^**^
Where to access screening	0.262 (0.408)	0.424 (0.266)	-0.193 (0.809)
Constant	-1.393 (0.051)^*^	-1.27 (0.136)	-1.953 (0.265)
Observations	500	413	87
Log likelihood	-97.222	-78.983	-14.823
Akaike information criterion	210.445	173.966	45.647

Barriers to colon-rectal screening with significant chi-square tests were also subjected to logistic regression (Table 3). The perception of colorectal not being a serious health threat increased the odds of the barrier to CR screening with the overall model (AdjOR = 2.059; 99% CI: 1.252-3.389), female participants (AdjOR = 1.834; 95% CI: 1.052-3.170), and male participants (AdjOR = 7.182; 95% CI: 1.292-66.472). However, lack of transportation increased the odds of the CR screening barrier only in the overall model (AdjOR = 1.589; 95% CI: 1.017-2.477).

**Table 3:** Barriers to colon rectal screening Significance levels: ^*^p < 0.1; ^**^p < 0.05; ^***^p < 0.01. The p values are enclosed in parentheses

Predictors	Overall model (1)	Females (2)	Males (3)
Is not mandatory	-0.182 (0.433)	-0.128 (0.609)	-1.463 (0.146)
Not effective	0.457 (0.077)^*^	0.406 (0.155)	0.474 (0.479)
Not a serious health threat	0.722 (0.005)^***^	0.606 (0.031)^**^	1.972 (0.044)^**^
Lack of transportation	0.463 (0.041)^**^	0.372 (0.146)	0.895 (0.106)
Constant	-6.006 (< 0.001)^***^	-5.512 (<0.001)^***^	-6.841 (-0.0002)^***^
Observations	500	413	87
Log likelihood	-146.112	-119.383	-24.017
Akaike information criterion	302.224	248.766	58.035

The reasons for the barriers toward colonoscopy were tested using the factors with significant chi-square test results. Overall, past bad colonoscopy experience increased the odds of not seeking CR screening in the overall model (AdjOR = 2.818; 99% CI: 1.751-4.640) and among female participants (AdjOR = 2.934; 99% CI: 1.692-5.239). The finding for male participants was statistically nonsignificant (Table 4).

**Table 4:** Barriers toward colonoscopy Significance levels: ^*^p < 0.1; ^**^p < 0.01. The p values are enclosed in parentheses

Predictors	Overall model (1)	Females (2)	Males (3)
Takes a lot of time	0.072 (0.797)	-0.010 (0.974)	0.318 (0.642)
It is not important	0.376 (0.139)	0.353 (0.237)	0.386 (0.431)
An expensive procedure	-0.177 (0.509)	-0.29 (0.336)	0.063 (0.919)
Past bad screening experience	1.036^**^ (0.00003)	1.076^**^ (0.0002)	0.910^*^ (0.071)
Constant	-5.790^**^ (< 0.001)	-5.314^**^ (< 0.001)	-6.715^**^ (0.001)
Observations	500	413	87
Log likelihood	-150.375	-118.458	-30.642
Akaike information criterion	310.751	246.916	71.284

The findings on the fecal occult blood test not being important increased the odds of CR screening in the overall model (AdjOR = 2.147; 99% CI: 1.374-3.423), among female participants (AdjOR = 1.810; 95% CI: 1.089-3.073), and male participants (AdjOR = 3.861; 99% CI: 1.514-11.673) (Table 5).

**Table 5:** Fecal occult blood test Significance levels: ^*^p < 0.1; ^**^p < 0.05; ^***^p < 0.01. The p values are enclosed in parentheses

Predictors	Overall model (1)	Females (2)	Males (3)
Is not mandatory	-0.182 (0.433)	-0.128 (0.609)	-1.463 (0.146)
Not effective	0.457^*^ (0.077)	0.406 (0.155)	0.474 (0.479)
Not a serious health threat	0.722^***^ (0.005)	0.606^**^ (0.031)	1.972^**^ (0.044)
Lack of transportation	0.463^**^ (0.041)	0.372 (0.146)	0.895 (0.106)
Constant	-6.006^***^ (<0.001)	-5.512^***^ (<0.001)	-6.841^***^ (0.0002)
Observations	500	413	87
Log likelihood	-146.112	-119.383	-24.017
Akaike information criterion	302.224	248.766	58.035

## Discussion

The present study found a strong association between knowledge of CR screening and CR screening among the general population in the Qassim region of the Kingdom of Saudi Arabia. People with a family history related to CR screening were about 15% more likely to know about CR screening than those without a family history. This phenomenon was profound among women, whose odds showed a reduced association, which was insignificant among men. This meant that a family history of CR screening would likely reduce awareness among the female participants. Guptaet al. [14] reported that early screening of CR based on family history shows increased chances of detecting and preventing CR cancer, meaning recommended screening initiation at an age younger than the observed age of diagnosis is appropriate. Likewise, the study by Fuchs et al. [15] reported an association between the risk of CRC and family history (odds 5.37) among young persons, and the risk decreased with increasing age, which is contrary to the National Cancer Institute [16], which reports increasing age is a major risk factor. However, its incidence increases among younger age groups. Colonoscopy around the time of first diagnosis should rule out synchronous neoplasms for surveillance of people at higher risk of colon cancer or rectal cancer that has been resected with curative intent.

Residents who had undergone symptom-based screening were almost twice as likely to know about CR screening, only among male participants. The clinical guideline and rationale for CRC screening and surveillance recommend that symptomatic individuals undergo appropriate diagnostic evaluation to distinguish them from asymptomatic individuals who are candidates for routine screening [17,18].

The role of health promoters in the contribution of knowledge about CR screening was very evident, as it showed that it made the residents about 35 times more likely to know about CR screening. Equally, it increased the likelihood of female participants by about 30 times, and male participants dramatically by about 241 times. This shows that the role of health promoters among the residents was very effective in increasing and promoting knowledge about CR screening among the residents across all groups, with the most effect being shown by the male residents. A tailored intervention increases the chances of an individual seeking a CR screening test ordered by a primary care provider [19]. Computer-delivered tailored interventions are more effective than a nontailored brochure when it comes to stimulating patient-provider communication about CR screening [19]. The delivery methods of CR screening awareness, educator authority, and educational content on screening behavior provided by trained academic health professionals are more effective in improving CRC screening rates than the tailored education provided by community health advisors [20]. A major challenge to CRC care will be doctors’ ignorance of future screening procedures and the projected impact of early FOBT screening in lowering mortality [21]. Studies by Alhuzaim et al. [8] and Alduraywish et al. [9] had previously reported fewer health check-ups and a lack of physician recommendations, respectively, which also contribute to barriers to CR screening.

Knowledge about methods of screening CRC was investigated, and the respondents were supposed to select all that apply. Colonoscopy represented 276 (29.9%), FOBT 173 (18.7%), and FIT 87 (9.4%). It is important to note that 116 (12.6%) of the residents were not aware of any of the above test methods. This meant that as the health facilities promoted awareness on matters of CR screening, there was likely to be a barrier to it.

The study also investigated barriers that would put the residents of Qassim in an awkward situation when it comes to seeking CR screening services from a health facility. Overall, the residents who perceived CR issues as not being a serious health threat were about two times more likely to face barriers to seeking CR screening. Although the perception was significantly reported among both genders, the effect was strong among male participants. This finding further reveals that female participants were 1.8 times more likely to face obstacles, though the impact was seven times stronger among male participants. Thus, perceiving CR screening as not being a serious health issue was a significant barrier to screening for both male and female participants, with the effect being much stronger among male participants. Lack of awareness about CRC risk and the perception that it is not a serious threat deters individuals from participating in CR screening programs [22].

Transportation to a health facility is very important when it comes to seeking health services. The present study reported that a lack of transportation was a barrier to seeking CR screening services, as it increased the likelihood of not seeking the services 1.6 times in the overall model. However, the study noted that the finding was not statistically significant when it came to segregating the data between male and female participants, as it was not affecting them differently. Distance to health facilities, transportation challenges such as lack of money to purchase fuel (gas), and lack of reliable vehicles pose significant challenges to accessing CR Screening services in rural communities [23,24]. Higher colonoscopy completion rates follow from reduced transportation barriers once suitable plans for transportation services are in place.

Past bad experiences in a facility when seeking certain health facilities can have some adverse impact. The present study investigated experiences associated with seeking colonoscopy health services. Residents with a past bad experience during colonoscopy were 2.8 times more likely to avoid seeking CR screening. This effect was stronger and was mainly observed among female participants, where such experiences made them about 2.9 times more likely to avoid CR screening in health facilities. However, it is important to note that a bad experience was a significant barrier to the overall model. The findings for the male residents were not statistically significant, which meant there was no clear evidence to indicate a relationship between past bad colonoscopy experiences and their decision to avoid CR screening. A physician’s study in New Mexico attributed the low uptake of CR screening to patient factors such as embarrassment, fear of pain, and lack of insurance [22]. Psychological factors, including fear of pain and discomfort of CR screening procedures (FIT screening), pose significant barriers to follow-up colonoscopy [25]. Negative experiences with healthcare services, including previous screenings, negatively impact participation in CRC screening [26], patient barriers, such as fear of pain, discomfort from previous procedures, apprehension over uncovering bad news about their health, and stigma associated with CR Screening [27].

Residents who believed the fecal occult blood test (FOBT) was unimportant were about 2.1 times more likely to undertake CR screening. Thus, findings show that residents who did not consider FOBT as important seem to be encouraged to undergo CR screening. The odds were slightly lower among the women, who were 1.8 times more likely to undergo CR screening, compared to the male participants, who were 3.9 times more likely to participate in CR screening. This means that women were more likely to seek CR screening if they perceived FOBT as less important, though the impact was smaller than for men. In the New Mexico study by Hoffman et al. [22], the respondents confirmed that the lack of physician discussion resulted in lower CR screening rates for both FOBT (45%) and endoscopy (34%), with the study also reporting asymptomatic increased chances of not seeking CR screening using FOBT (22%) and endoscopy (36%).

The present study investigated factors that might prevent the residents from having CR screening, and multiple responses were allowed. The top three factors were fear and anxiety about CR screening, representing 222 (25.3%), lack of information, 166 (18.9%), and cost, 119 (13.6%). Others were lack of time 110 (12.5%), inconvenience of scheduling 101 (11.5%), lack of insurance 54 (6.2%), and difficulty scheduling an appointment 17 (1.9%). However, it was important to note that 88 (10%) of the respondents noted other factors, which clearly indicates that there were other factors beyond the focus of the present study that would prevent the residents from seeking CR screening. A lack of awareness [4,22], fear of finding cancer [4,22], and embarrassment/anxiety about testing [6,22] have been reported as barriers to CR screening. The absence of symptoms is likely to push individuals to perceive CRC screening as unnecessary, contributing to low uptake of FOBT [28]. Fecal occult blood testing has been perceived as distasteful, and thus most individuals may not perceive its importance, thus posing a significant barrier to screening [21]. Individual attitudes, lower perceived disgust, higher socioeconomic status, perceived ease of completion, and previous participation in any cancer screening are predictors of intention to participate in CRC screening programs and complete the FOBT [29].

Among the residents who had previously undergone CR screening, the most popular methods they used were colonoscopy, 37 (11.2%), FOBT, 24 (7.3%), and CT colonography (virtual endoscopy), 21 (6.3%). On the same note, those who sought sigmoidoscopy were 10 (3%), stool DNA test 9 (2.7%), and FIT 7 (2.1%). However, a major gap was identified, representing 223 (67.4%) of the residents who did not select any of the listed tests. Thus, future studies should be designed to allow the residents a scenario where they can state the test they knew about.

One of the limitations of this study was the strong skew in the data toward female patients, with 413 (82.6%) compared to 87 (17.4%) male patients. This imbalance may affect the generalizability of the findings. However, the gender-segregated analysis provided valuable comparative insights, revealing significant differences between the two groups. This suggests that achieving a balanced representation of both genders is essential for meaningful comparisons. Nevertheless, CRC may be perceived differently by male and female participants, potentially influencing the uptake of related healthcare services.

Although the present study did not investigate any cultural factors, there could be taboos associated with certain medical check-ups that might be considered intrusive to privacy among Saudis. A study by Alduraywish et al. [9] in Saudi Arabia associated hesitancy toward CRC screening among many Saudis with the procedure being painful, humiliating, and a cause of unnecessary anxiety. Nevertheless, some medical tests could be viewed as intrusive by individuals with strong cultural backgrounds and traditions that place great significance on certain parts of the body considered private. Female participants may be more accustomed to medical examinations involving private body parts compared to male participants, such as antenatal and hospital-based delivery-related examinations. Therefore, future studies are recommended to include the cultural aspect of Saudi society to investigate its impact on gastrointestinal health issues, which may be perceived as unsuitable for public discussion.

Practical implications and recommendations

Knowledge of CRC screening, family history, and awareness of CR screening identified a gap in awareness levels among female participants, which can be bridged through targeted efforts across all genders. Symptom-based screening increased knowledge of CR screening among male participants, highlighting an opportunity that can be effectively utilized to educate men about CR screening methods. Knowledge of CR screening increases when awareness programs and promotions are conducted by health promoters.

On barriers to the uptake of CR screening, the perception that CR is not a serious health threat, especially among male participants, needs to be addressed to promote behavioral change by demystifying misconceptions about CR screening. Transportation challenges pose barriers to seeking CR screening, highlighting the need for transportation solutions to improve access to CR screening facilities. Previous negative experiences related to colonoscopy discourage CR screening, particularly among female participants. Therefore, health facilities and personnel need to design awareness programs to improve the quality of care and provide positive experiences during screening procedures. Educational campaigns should be increased to highlight the role of each screening method and ensure they align with patients' screening behaviors, as the present study indicated that more men were likely to undergo other CR screening methods once they perceived FOBT as less important. Psychological support, public education, and financial assistance can help reduce barriers to CR screening, such as fear and anxiety, lack of information, and cost implications. Finally, most Qassim residents have expressed a significant knowledge gap regarding specific screening methods, underscoring the need for expanded programs on CR screening awareness and behavior change.

## Conclusions

The study reveals in the Qassim Region notable information gaps on CR screening and low awareness of screening techniques. A family history of CR screening is associated with reduced awareness among female participants. Health promoters significantly increase CR screening awareness among residents. Improved patient experience, transportation support, better understanding and techniques for CR screening, explanation of CR screening techniques, removal of emotional obstacles, and lowering of financial constraints, can help increase the acceptance of CR screening programs and awareness of CR techniques.

## Appendices

The study tool

Demographic Information

1. Age

2. Sex

3. Level of Education

An Assessment of Patient Knowledge About Colorectal Cancer Screening

1. Have you heard about early screening for colorectal cancer?

2. How important do you think colorectal cancer screening is for early detection of cancer?

3. Which of the following methods of screening for colorectal cancer do you know? (Select all that apply)

4. At what age do you think colorectal cancer screening should begin for individuals who do not have risk factors (such as a family history)?

5. How often do you think a person should be screened for colorectal cancer?

6. Do you think a family history of colorectal cancer affects the need for early screening?

7. Do you think colon cancer screening is only necessary if you have symptoms?

8. Have you ever discussed colorectal cancer screening with your health care provider?

9. Do you know where you can get a colon and rectal cancer screening?

10. What factors might prevent you from having colorectal cancer screening? (Select all that apply)

An Assessment of Previous Experience With Screening

11. Have you ever had any type of colon or rectal cancer screening?

12. If no, have you considered screening for early detection of colorectal cancer?

13. If yes, what method did you use?

14. If yes, how would you rate your experience with colon and rectal cancer screening?

An Assessment of the Barriers to Colorectal Cancer Screening

15. Which of the following options prevents you from getting colorectal cancer screening? (I believe CRC screening is not effective)

16. Which of the following options prevents you from getting colorectal cancer screening? (Colorectal cancer is not a serious health threat)

17. Which of the following options would prevent you from having a colorectal cancer screening? (It is difficult to get an appointment with a physician)

18. Which of the following options would prevent you from having a colorectal cancer screening? (I don’t have a physician’s recommendation for CRC screening)

19. Which of the following options would prevent you from having a colorectal cancer screening? (I don’t have any symptoms of getting screened for CRC)

20. Colonoscopy-related barriers (Colonoscopy takes a lot of time)

21. Colonoscopy-related barriers (A colonoscopy isn’t important, in my opinion)

22. Barriers to colonoscopy (Colonoscopy is an expensive procedure)

23. Colonoscopy-related barriers (I think colonoscopy is very painful)

24. Colonoscopy-related barriers (Colonoscopy is a very embarrassing procedure)

25. Colonoscopy-related barriers (I am afraid of the results of the colonoscopy)

26. Colonoscopy-related barriers (I am afraid of colonoscopy complications)

27. Colonoscopy-related barriers (I had a previous bad experience with colonoscopy)

28. Colonoscopy-related barriers (I don’t know where I can get a colonoscopy)

An Assessment of the Barriers to FOBT

29. Cons of fecal occult blood testing (FOBT) (I think a fecal occult blood test, FOBT, isn’t important)

30. Barriers to fecal occult blood testing (FOBT) (FOBT is an expensive procedure)

31. Barriers to fecal occult blood testing (FOBT) (I don’t have time to get a test for FOBT)

32. Barriers related to fecal occult blood testing (FOBT) (I am afraid of the results of the FOBT)

33. Barriers to faecal occult blood testing (FOBT) (I am feeling bad about getting the FOBT done)

34. Barriers to fecal occult blood testing (FOBT) (I am feeling bad about getting the FOBT done)
